# Role of Histone Deacetylases in Skeletal Muscle Physiology and Systemic Energy Homeostasis: Implications for Metabolic Diseases and Therapy

**DOI:** 10.3389/fphys.2020.00949

**Published:** 2020-08-11

**Authors:** Haili Tian, Sujuan Liu, Jun Ren, Jason Kai Wei Lee, Ru Wang, Peijie Chen

**Affiliations:** ^1^School of Kinesiology, Shanghai University of Sport, Shanghai, China; ^2^Department of Anatomy and Histology, School of Basic Medical Sciences, Tianjin Medical University, Tianjin, China; ^3^Department of Cardiology, Shanghai Institute of Cardiovascular Diseases, Zhongshan Hospital Fudan University, Shanghai, China; ^4^Department of Physiology, Yong Loo Lin School of Medicine, National University of Singapore, Singapore, Singapore; ^5^Global Asia Institute, National University of Singapore, Singapore, Singapore; ^6^N.1 Institute for Health, National University of Singapore, Singapore, Singapore

**Keywords:** histone deacetylases, exercise capacity, skeletal muscle, metabolism, muscle physiology

## Abstract

Skeletal muscle is the largest metabolic organ in the human body and is able to rapidly adapt to drastic changes during exercise. Histone acetyltransferases (HATs) and histone deacetylases (HDACs), which target histone and non-histone proteins, are two major enzyme families that control the biological process of histone acetylation and deacetylation. Balance between these two enzymes serves as an essential element for gene expression and metabolic and physiological function. Genetic KO/TG murine models reveal that HDACs possess pivotal roles in maintaining skeletal muscles’ metabolic homeostasis, regulating skeletal muscles motor adaptation and exercise capacity. HDACs may be involved in mitochondrial remodeling, insulin sensitivity regulation, turn on/off of metabolic fuel switching and orchestrating physiological homeostasis of skeletal muscles from the process of myogenesis. Moreover, many myogenic factors and metabolic factors are modulated by HDACs. HDACs are considered as therapeutic targets in clinical research for treatment of cancer, inflammation, and neurological and metabolic-related diseases. This review will focus on physiological function of HDACs in skeletal muscles and provide new ideas for the treatment of metabolic diseases.

## Introduction

Skeletal muscle is the largest metabolic organ in the human body, consuming about 18% of the entire body daily expenditure of energy (Durnin, 1981). It produces various secrete factors and participates in the interplay among multiple tissues and organs (Pedersen and Febbraio, 2012; Mizgier et al., 2019). Muscular contraction is one of the main physiological functions of skeletal muscles and plays a role in maintaining organ and systemic metabolic homeostasis (Guo et al., 2020). Proper exercises help build up body defense to combat various diseases including obesity, type 2 diabetes, Alzheimer disease, osteoarthritis, and so on (Luan et al., 2019). These important functions require delicate regulations in muscle from the level of genome to signal transduction, establishing muscle plasticity, and responses to environmental stress. Acetyl-CoA as a central metabolite of amino acid/fatty acid/glucose not only serves as fuel for energy expenditure but also bridges the gap between environmental stress and organismal response (Lempradl et al., 2015; Liu et al., 2020). This control occurs through post-translational modification on nucleosomes histone lysine residues and other signal transducing proteins, which in turn controls accessibility of DNA to regulatory factors and protein activity. Acetylation, one of the most prevalent modifications of protein, is believed to function as a key modulator of chromatin structure and signal transduction, and provides an avenue to couple extracellular stimuli with genome during muscle metabolism process by regulating acetylation and deacetylation (Haberland et al., 2009).

histone deacetylases (HDACs) as a family of protein deacetylases have been demonstrated to moderate physiological homeostasis and development by deacetylation (Haberland et al., 2009) (Table 1). In humans, there are 18 types of HDACs, which are classified into four categories based on homologous proteins in yeast, namely: Class I Rpd3-like protein (HDAC1, -2, -3, -8); Class II Hda1-like proteins are classified as Class IIa (HDAC4, -5, -7, -9) and Class IIb (HDAC6, -10); Class III Sir2p-like, nicotinamide adenine dinucleotide (NAD^+^)-dependent (SIRT1, -2, -3, -4, -5, -6, -7); and Class IV HDAC11, containing homologous domain with both Rpd3 and Hda1 (Seto and Yoshida, 2014). HDACs modulate gene expression and protein activity through deacetylating proteins. When histone lysine ε-amino acid in the nucleosome is acetylated, it can neutralize positive charge, before loosening chromatin structure, to promote binding of transcription factors to DNA and expression of downstream target genes. By contrast, histone deacetylation compresses chromatin structure, thereby inhibiting transcriptional gene expression (Shahbazian and Grunstein, 2007).

**TABLE 1:** The targets of HADCs in skeletal muscles and their physiological functions.

Class	Members	Down targets	Genetic KO/TG phenotype	References
I	HDAC1	MyoD↓, *PTEN*↓, FoxO?	Functionally redundant HDAC1/2 DKO mice causes mitochondrial abnormalities and sarcomere degeneration	Puri et al., 2001; Ohkawa et al., 2006; Beharry et al., 2014; Cho et al., 2015; Zhu et al., 2016; Bin et al., 2019
	HDAC2	MyoD↓, MEF2D↓, *NF-*κ*Bp65*↓		
	HDAC3	MEF2D, PCAF↓, *Ampd3*↓ RCAN1? MEF2A-*Cpt1b*↓	mKO mice: Enhanced amino acid\lipid metabolism and endurance exercise Causes IR under basal condition	Gregoire et al., 2007; Han et al., 2014; Yuan et al., 2014; Hong et al., 2017
	HDAC8	–	–	
IIA	HDAC4	MEF2↓, *Glut4*↓, Myosin Heavy Chain, PGC-1α, and Hsc70-, *Pax7*↓	Functionally redundant HDAC4/5 DKO, HDAC5/9 DKO, HDAC4/5/9 TKO increases type I fiber percentage and oxidation metabolism	McKinsey et al., 2000a; Choi et al., 2014; Niu et al., 2017; Luo et al., 2019
	HDAC5	MEF2↓, *Glut4*↓, *Baf60c*↓		Yuan et al., 2014; Meng et al., 2017; Niu et al., 2017
	HDAC9	*Atg7, Beclin1, LC3*↓ Dach2-*Myog, Gdf5*↓		Macpherson et al., 2015; Zhang et al., 2019
	HDAC7	MEF2↓	–	Dressel et al., 2001
IIB	HDAC6	*Pax7*, MFN1↓ Fam65b, dysferlin and MAFbx-	KO mice: Causes mitochondrial oxidative damage, mitofusion defect Protect against muscle wasting	Balasubramanian et al., 2014; Lee et al., 2014; Ratti et al., 2015
	HDAC10	–	–	
III	SIRT1	PGC-1α? STAT3↓	mKO mice: Mediates CR induced insulin sensitivity, has no effect on glucose homeostasis or exercise capacity TG mice: no effect on glucose homeostasis or exercise capacity	Rodgers et al., 2005; Schenk et al., 2011
	SIRT2	–	KO mice: Exacerbates obesity and IR in HFD	
	SIRT3	MnSOD?, Hexokinase II? PDH subunit E1α?	KO mice: Exacerbates obesity and IR in HFD	Boyle et al., 2013; Jing et al., 2013; Lantier et al., 2015
	SIRT4	Malonyl CoA decarboxylase↓	KO mice: Increased exercise tolerance and protect against HFD-induced obesity	Laurent et al., 2013
	SIRT5	–	–	
	SIRT6	NF-κB-*Mstn*	KO mice: Abnormal hypoglycemia TG mice: Protect against HFD mKO mice: Impaired insulin sensitivity, loss of muscle mass	Kanfi et al., 2010; Xiao et al., 2010; Cui et al., 2017; Samant et al., 2017
	SIRT7	–	–	–
IV	HDAC11	–	–	–

*Activation?, inhibition↓, unknown. KO, whole-body deletion; TG, whole-body overexpression; DKO, double knockout; TKO, triple knockout; mKO, muscle-specific knockout; CR, caloric restriction; HFD, high fat diet; IR, insulin resistance.*

Biochemical properties of HDACs have been comprehensively reviewed (Shahbazian and Grunstein, 2007). However, the physiological functions of HDACs have yet to be well examined in skeletal muscles. A series of researches shed light on the potential of drugs targeting HDACs to improve muscle fitness and cardiac muscle disease, which needs further clarification of HDACs physiological function to understand the mechanisms (Bai et al., 2011; Galmozzi et al., 2013; Xie et al., 2014; Zhang and Ren, 2014; Gaur et al., 2016). Ample work has revealed the role of HDAC in muscle physiology such as skeletal muscles me olism and thus exercise capacity (McGee and Hargreaves, 2010) (Table 1). Class I HDACs can interact with myocyte enhancer factor 2 (MEF2), MyoD, regulating myogenesis, and exercise capacity (Mal et al., 2001; Puri et al., 2001; Ohkawa et al., 2006; Gregoire et al., 2007). HDAC3 participates in myocytes differentiation and is linked to skeletal muscles metabolic fuel switching (Gregoire et al., 2007; Hong et al., 2017). Class II HDACs can be phosphorylated by HDAC kinases and are transduced from nucleus to cytoplasm in response to cellular stress, mediating muscle fiber switch and affecting the pathway of insulin sensitivity such as *Glut4* and AKT (Haberland et al., 2009; McGee and Hargreaves, 2010). Class III HDACs target a crucial mitochondrial biogenesis factor peroxisome proliferator-activated receptor gamma, coactivator 1 alpha (PGC-1α) in skeletal muscles, liver and fat tissue (White and Schenk, 2012). Therefore, we will further discuss how these HDACs influence respective downstream targets, exercise capacity, and therapeutic effects in human diseases.

## Class I HDAC: HDAC1/2/3

### Regulation of Myogenesis by Class I HDACs

**FIGURE 1:**
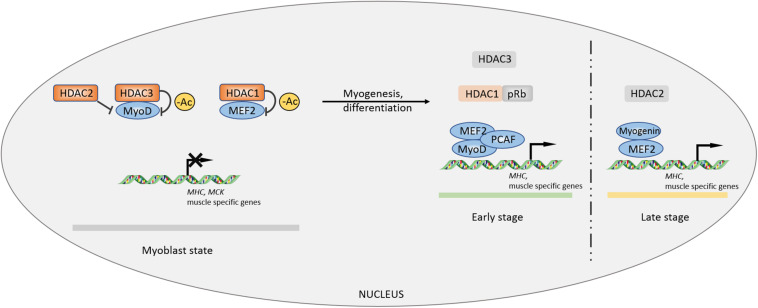
Controls of myogenesis by Class I HDACs. The inhibitory effects of HDACs on myogenic factors MEF2 and MyoD maintain a primary myoblast state. During myocyte differentiation and myogenic process, the inhibition is lifted by other factors, facilitating MEF2-MyoD complex formation to promote myogenesis. –Ac, deacetylation.

In previous studies, HDAC1/2/3 was shown to be associated with the process of myogenesis or myocyte differentiation (Mal et al., 2001; Puri et al., 2001; Ohkawa et al., 2006; Gregoire et al., 2007). HDAC1 tightly binds to MyoD and deacetylates specific sites to inhibit the expression of muscle-specific genes such as *MHC* and *MCK* in myoblasts. Mimicking muscle differentiation condition by serum deprivation, HDAC1 protein gradually decreases during myocyte differentiation and transfers to bind to the tumor suppressor pRb, accompanied by isolation of HDAC1-MyoD and transcriptional activation of muscle-specific genes (Puri et al., 2001). A HDAC1 (H141A) mutant incapable of binding with MyoD loses its inhibitory property on muscle-specific genes in myoblast state (Mal et al., 2001). In the late stage of myocyte differentiation, activating factor gradually switches from MyoD to myogenin, occurring with a decrease in the MEF2D inhibitory regulator HDAC2. Simultaneous overexpression of myogenin and MEF2D can enhance the expression of the muscle-specific gene *MHC* in the absence of MyoD (Ohkawa et al., 2006). MEF2D, a key factor that controls myocyte differentiation, can only be effectively deacetylated by HDAC3, rather than HDAC1/2/8 (Gregoire et al., 2007). In addition, HDAC3 can inhibit autoacetylation of acetyltransferases p300 and p300/CBP-associated factor (PCAF). Thus, HDAC3 impedes MyoD-MEF2-PCAF to form a multicomplex, disturbing MEF2-dependent myogenic transcription (Gregoire et al., 2007; Figure 1).

### Redundant Roles of HDAC1/2 in Maintaining Sarcomere Homeostasis in Muscle

Loss of function of HDAC1/2 inhibits autophagy flux in skeletal muscles tissue, accompanied by decreased LC3 II/I ratio and p62 accumulation under fasting condition (Moresi et al., 2012). Toxic autophagy intermediates accumulate in muscle fibers in which class I HDACs deficiency led to impaired exercise capacity (Moresi et al., 2012). This offers convincing evidence on the role of HDAC1/2 in stabilizing basic structure and facilitating development in muscles. Genetic models suggest that Class I HDACs control the physiological homeostasis of skeletal muscles. A germline deletion shows that whole-body knockout of either HDAC1 or HDAC2 leads to mortality in mice prior to the perinatal period (Montgomery et al., 2007). However, a tissue-specific single deletion of HDAC1 or HDAC2 in myocardium does not cause any phenotype (Montgomery et al., 2007). Double knockout HDAC1/2 in the heart leads to severe cardiomyopathy in mice, indicating the obligatory role for HDAC1/2 in specific tissues (Montgomery et al., 2007). In skeletal muscle, double knockout of HDAC1/2 by myogenin-Cre causes mitochondrial abnormalities and sarcomere degeneration, disrupting fundamental structural units of myofibers (Moresi et al., 2012). Therefore, these findings demonstrate that HDAC1/2 redundantly maintains sarcomere homeostasis in skeletal muscle.

### HDAC3 Functions as a Fuel Switch in Muscle Energy Metabolism

Histone deacetylases 3 is the enzymatic core of nuclear receptor corepressor (N-CoR) and silencing mediator of retinoic acid and thyroid hormone receptors (SMRT) co-repressor; correspondingly, N-CoR and SMRT co-repressor activate HDAC3 through its SANT domain enzyme activity (Karagianni and Wong, 2007). Mice suffer from insulin resistance after specifically knocking out HDAC3 in skeletal muscles-mKO, while their endurance exercise abilities are enhanced (Hong et al., 2017). It seems a self-contradictory phenomenon because former studies pointed out that increased endurance exercise capacity enhances insulin sensitivity. The study shows that insulin signaling cascades including pAKT/AKT, pIRS1-S1101, and pGSK/GSK have no change in HDAC3 mKO muscle, while glucose uptake and insulin sensitivity are impaired in glucose tolerance test (GTT) and insulin tolerance test (ITT) (Hong et al., 2017), which is called the “dissociation effect” by researchers. Lipid tends to be used as energy fuel in HDAC mKO mice, which inhibits glucose absorption but does not influence insulin signaling sensitivity (Hong et al., 2017). Further work found that the HDAC3 knockout upregulates the expression of AMP deaminase 3 (AMPD3), the first rate-limiting enzyme in purine metabolism, in skeletal muscles (Fortuin et al., 1996; Hong et al., 2017). AMPD3 can deaminate AMP to form IMP, facilitating aspartic acid to transmit into fumarate and malate, the intermediate metabolites of tricarboxylic acid cycle (TCA cycle) (Hong et al., 2017). Research studies found that exercise induced glucose labeled 13C6 expressed a lower glycolysis flux rate in muscles of HDAC3 mKO but a higher expression in the TCA cycle intermediates (Hong et al., 2017; Gong et al., 2018). Thus, a general increase in TCA cycle metabolites activates the oxidation in mitochondria. *In vitro* radioactive aspartic acid isotope tracer test showed that inhibiting HDAC3 or overexpressing AMPD3 can increase amino acid metabolism rate and enhance fatty acid metabolism, thereby downregulating glucose metabolism (Hong et al., 2017Figure 2). In liver, knockout HDAC3 causes severe liver steatosis and can be rescued by wild-type or catalytically inactive mutants of HDAC3, indicating an enzymatic activity independent function (Sun et al., 2013). However, the muscle fuel switching in HDAC3 mKO mice cannot be rescued by an enzymatic inactivity mutant HDAC3 (Song et al., 2019). This finding demonstrates that HDAC3 controls the fuel utilization in skeletal muscles and is dependent on its enzymatic activity. The relationship between exercise and glucose uptake is far more complex than one could expect.

**FIGURE 2:**
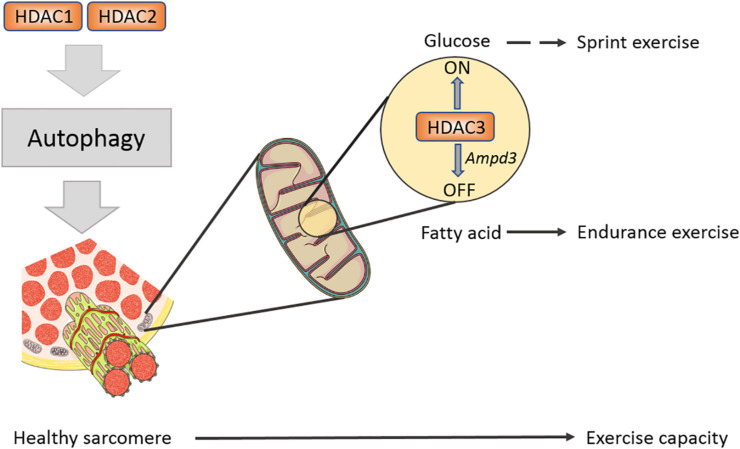
Class I HDACs regulates sarcomere structure and exercise capacity in mature muscle. A schematic diagram shows Class I HDACs regulate exercise capacity on different levels. HDAC1/2 have redundant roles in modulating autophagy flux in muscle, eliminating toxic factors in muscle. HDAC3 serves as a fuel switch and promotes muscle to use fatty acid to adapt endurance exercise.

## Class II HDACs: HDAC4/5/7/9/6

### Phosphorylation and Nucleus-Cytoplasm Shuttle of Class II HDACs

The MEF2 family is a class of transcription factors that are closely associated with myogenic basic helix-loop-helix domains, including MyoD (Taylor and Hughes, 2017). MEF2 may interact with Class IIa histone deacetylase HDAC4/5 to inhibit the transcription of MEF2-dependent genes, thereby impeding the differentiation from myoblast to myotube (McKinsey et al., 2000a). During exercise, Ca^2+^ flows out from sarcoplasmic reticulum and activate calcium/calmodulin-dependent protein kinase (CaMK), which phosphorylates HDAC4/5 and mediates HDAC4/5 shuttle from the nucleus to the cytoplasm, thus releasing the repressive effect of HDAC4/5 on MEF2-dependent genes (McKinsey et al., 2000b; Yuan et al., 2014). Further research demonstrates that the nucleoplasmic shuttle of HDAC4 relies on 14-3-3 binding, while the shuttle of HDAC5 depends on CaMK phosphorylation at serine -259 and -498 sites first, and then exporting from the nucleus by binding with 14-3-3 (McKinsey et al., 2000b). As expected, HDAC7 in Class IIa also possesses the ability to be exported from nucleus to cytoplasm (Gao et al., 2010), suggesting that there may be redundant functions of Class IIa HDACs. Except for Class IIa, knocking out Class IIb HDAC6 in embryonic stem cells (ESCs) can promote ESCs to differentiate into myoblast cells, and transplantation of ESCs with HDAC6 knockdown can also help the regeneration of wound skeletal muscles (Lee et al., 2015).

### Regulation of Class II HDACs on Fiber-Type Switch, Mitochondria Remodeling, and Energy Stress in Skeletal Muscle

**FIGURE 3:**
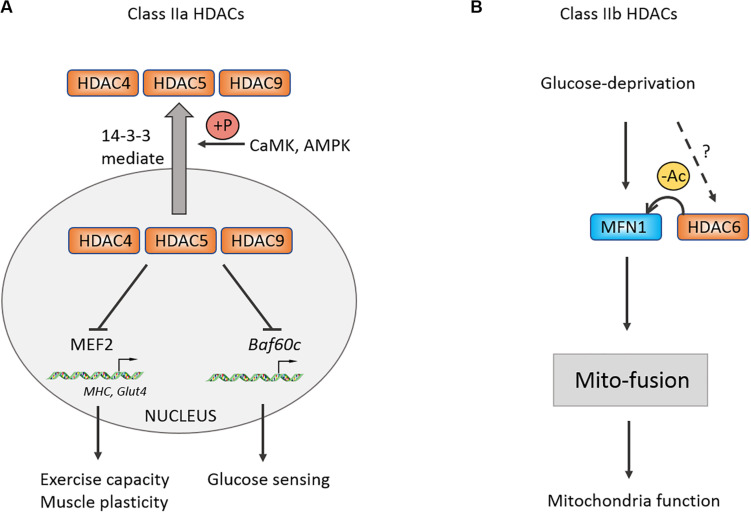
Control of muscle exercise adaption and mitochondrial function by Class II HDACs. **(A)** Schematic of the Class IIa HDACs nucleoplasm shuttle. Under stress condition, CaMK and AMPK could be activated and phosphorylate Class IIa HDACs. Phosphorylated HDACs then bind with 14-3-3 and shuttle to cytoplasm, relieving inhibitory effects on MEF2 and *Baf60c*. **(B)** Class IIb HDAC6 can deacetylate MFN1 and facilitates its mito-fusion function. +P, phosphorylation.

Evidence shows that the proportion of type I fiber in skeletal muscles has no change in animal knocked out alone any member of Class IIa (Potthoff et al., 2007). However, the proportion of type I fiber is significantly increased in HDAC4/5 DKO, HDAC5/9 DKO, HDAC4/5/9 TKO mice as well as exercise capacity of skeletal muscles (Potthoff et al., 2007). Altogether, the finding indicated a functional redundant mechanism in Class II HDACs family. Previous study found that Class IIa HDACs are closely associated with the MEF2 family and can repress their downstream target genes (McKinsey et al., 2000a). Therefore, similar to Class IIa HDACs DKO, the proportion of type I fiber is decreased in the MEF2C and MEF2D knockout mouse models, overexpressing MEF2C results in increasing type I fiber and exercise capacity. HDAC4/5 not only regulates exercise capacity but also governs motor adaptation. Several studies had shown that exercise promotes phosphorylation of HDAC4/5 mediated by CaMK and adenosine monophosphate-activated protein kinase (AMPK), then increasing a MEF2-dependent transcription of *Glut4* and muscle-specific genes, which participates in the adaptation and plasticity of skeletal muscles (McGee et al., 2008, 2009; McGee and Hargreaves, 2011). Besides exercise, glucose can also activate K_ATP_ channel-dependent calcium signaling and CaMK, which induce phosphorylation-dependent nuclear-cytoplasm transportation of HDAC5 (Meng et al., 2017). The leaving HDAC5 releases its repressive effect on *Baf60c* and enhancing insulin-independent AKT activation (Meng et al., 2017). Using specific inhibitor of class IIa HDAC activity, myosin heavy chain, PGC-1α, and heat-shock cognate (HSC70) are confirmed to be one of the substrates of HDAC4 in denervation-induced atrophy muscle in comparison with acetylated protein enrichment with untreated group (Luo et al., 2019). Taken together, Class IIa HDACs play critical roles in exercise capacity, remodeling, and nutrient sensing of skeletal muscles (Figure 3).

For Class IIb, it was revealed that glucose deprivation-induced mitochondrial fusion mediated by mitofusion1 (MFN1) is significantly disrupted in HDAC6 KO mice, causing mitochondria degeneration, which is reversed following application of MFN1 acetylation-resistant mutant (Lee et al., 2014). This study suggested that MFN1 deacetylation by HDAC6 plays an important role in mitochondrial adaptive energy production and remodeling of skeletal muscles.

## Class III HDACs/Sir2-Homolog: SIRT1/6/3/4

### Nuclear-Located SIRT1/6

Numerous studies have found that caloric restriction (CR) extends lifespan of mammals, *Caenorhabditis elegans*, and fruit flies, while such effect is lost in *SIR2* or *NPT1* mutant *C. elegans* strains (Imai et al., 2000; Lin et al., 2000). *SIR2* encodes the silencing protein, Sir2p, and NPT1 participates in the synthesis of NAD^+^. NAD^+^ is an essential cofactor for Sir2p-Class III HDACs to exert enzyme activity rather than Class I, II, and IV relaying on the zinc (Imai et al., 2000; Lin et al., 2000). In mammals, Sir2 has 7 homologous proteins and these proteins are essential to mitochondrial energy homeostasis, antioxidant defense, cell proliferation, and DNA repair (Vargas-Ortiz et al., 2019).

#### SIRT1 Mediates Energy Stress Adaptation in Skeletal Muscles

**FIGURE 4:**
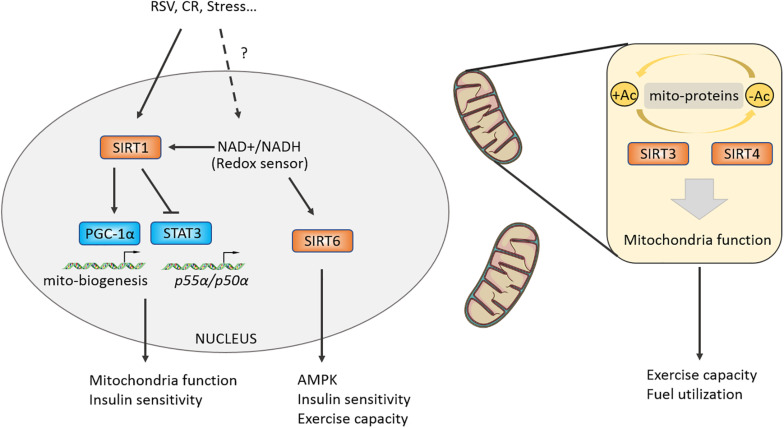
Class III HDACs promote a stress-induced adaptation in muscle and regulate mitochondrial function. Class III HDACs are NAD^+^-dependent deacetylases and may function as a redox sensor in muscle cell. SIRT1 could activate PGC-1alpha by directly deacetylasing K13 site and regulate insulin sensitivity by targeting STAT3. Mitochondria-located Class III HDACs control mitochondrial function by deacetylating certain mitochondrial proteins, modulating muscle fuel utilization and exercise capacity.

Rodgers et al. (2005) reported that SIRT1 promotes the transcriptional activity of PGC-1α by deacetylating the PGC-1α at K13 site and mediates CR-induced gluconeogenesis-related genes *G6P*, *PEPCK* expression in liver. Further research showed that the NAD^+^/NADH ratio and the enzymatic activity of Sir2, a sensor of redox state, were decreased in differentiating myocytes, which relieved the inhibitory effect on MyoD and then promoted cell differentiation. Resveratrol (RSV), a compound that may target SIRT1-PGC-1α, can improve mitochondrial function and metabolic homeostasis, owning a potential to prolong lifespan. RSV loses its activating effect on PGC-1α in SIRT1 (^–/–^) MEFs (Lagouge et al., 2006). Moreover, RSV-treated mice at a dose of 4 g/kg increased their exercise capacity, oxygen consumption, and improved insulin sensitivity against high-fat induced obesity (Lagouge et al., 2006). In addition, RSV could enhance the deacetylation of PGC-1α in brown fat tissue (BAT) and skeletal muscles, which promotes mitochondrial production, oxygen consumption, and thus improves metabolic syndrome (Lagouge et al., 2006). The function of SIRT1 in regulating skeletal muscles metabolic homeostasis and exercise capacity has attracted great attention from researchers. Researchers further generated skeletal muscle-specific SIRT1-mKO mice and found that mKO mice lost the effect of CR-induced increasing insulin sensitivity and were unable to deacetylate and inactivate STAT3, resulting in upregulating the phosphatidylinositol-3-kinase (PI3K) inhibitory regulator p55α/p50α expression (Schenk et al., 2011). Although knockout or overexpression of SIRT1 in skeletal muscles has no effect on endurance capacity or glucose homeostasis in mice (Gurd et al., 2009; Philp et al., 2011; White et al., 2013; Svensson et al., 2020), some studies have shown that AMPK regulates energy metabolism of skeletal muscles partially mediated by SIRT1, and SIRT1 is significantly increased after endurance exercise (Suwa et al., 2008; Canto et al., 2009). Overexpression of Sirt1 in skeletal muscles by adeno-associated virus 1 (AAV1) promotes the expression of oxidation-related genes including *Ppargc1a*, *Tfam*, *Cpt1b*, and *Pdk4*, while it has no effect on insulin sensitivity of body (Vila et al., 2016). But overexpressing Sirt1 in liver by AAV8 can protect from fatty liver induced by high-carbohydrate food (HCD) (Vila et al., 2014). Taken together, the aforementioned studies suggest that SIRT1 possesses limited regulation capacity of skeletal muscles motility, and SIRT1 may modulate mitochondrial homeostasis and mediate skeletal muscles adaptation under certain physiological conditions, such as CR, aging, and regeneration (Gomes et al., 2013; Ryall et al., 2015; Figure 4). In addition, there may be more beneficial effects of SIRT1 on metabolism in the liver or fat tissue (Lagouge et al., 2006; Vila et al., 2014, 2016; Stefanowicz et al., 2018).

### SIRT6 Improves Muscle Fitness and Exercise Capacity

SIRT6 is another Sir2-like deacetylase located in the nucleus. Whole body deletion of SIRT6 causes 60% mice death around 4 weeks owning to hypoglycemia (Xiao et al., 2010). Nevertheless, the skeletal muscle-specific knockout of SIRT6 causes insulin resistance and impairs glucose homeostasis of mKO mice (Cui et al., 2017). Because the activity of AMPK is reduced in SIRT6-mKO mice, the glycolipids absorption and utilization of skeletal muscles are impaired, and the exercise capacity of the mice was also attenuated (Cui et al., 2017). Furthermore, researchers constructed the overexpressing SIRT6 (Sirt6BAC) mice and found that their body weight and fat content were normal, but the Sirt6BAC mice could protect from HCD-induced hyperglycemia via increasing p-AKT/AKT induced by insulin stimulation and glucose absorption (Anderson et al., 2015). Vitro experiments further demonstrated that the ability of glucose uptake was enhanced mainly in skeletal muscles, not in brain, iWAT, eWAT tissues of Sirt6BAC mice (Anderson et al., 2015). These results shows the potential effects of SIRT6 targeted in skeletal muscles on improving exercise performance and treating metabolic diseases (Figure 4).

### Mitochondria-Located SIRT3/4

#### SIRT3/4 Maintains Mitochondria Function in Skeletal Muscles

SIRT3/4 are mitochondria-localized deacetylases and responsible for regulating the deacetylation of proteins in mitochondria and maintaining mitochondrial homeostasis. After feeding high-fat-diet (HFD), accelerated obesity, attenuated insulin sensitivity, worsened fatty liver and increased acetylation of proteins in mitochondria were observed in SIRT3 KO mice (Hirschey et al., 2011). Multi-tissue proteomics and physiological examination reveals that SIRT3 is responsible for mitochondrial acetylated proteome regulation and metabolic fuel switching in brain, heart, kidney, liver, and skeletal muscles (Dittenhafer-Reed et al., 2015). Protein enrichment analysis discovers that its regulatory proteins were mainly concentrated in lipid metabolism (Dittenhafer-Reed et al., 2015). Knocking out SIRT3 leads to reduction in the levels of deacetylation of manganese superoxide dismutase (MnSOD) mitochondrial complex II and pyruvate dehydrogenase (PDH) subunit E1α, a downregulation of hexokinase II (HK II) binding with mitochondria, resulting in impaired glucose and lipid metabolism of skeletal muscles (Lantier et al., 2015). Interestingly, SIRT3 liver-specific knockout (hep^–/–^) and skeletal muscle-specific knockout (skm^–/–^) mice did not affect glucose homeostasis under chow or HFD conditions (Fernandez-Marcos et al., 2012). The above results indicate that SIRT3 has an important role in metabolic regulation, but in specific physiological processes such as redox state, exercise, and aging, and its regulatory mechanism is yet defined in skeletal muscles (Kong et al., 2010; Robin et al., 2020; Williams et al., 2020). A recent study found that SIRT4 knockout can resist HFD-induced obesity and increase endurance exercise in mice by repressing malonyl CoA decarboxylase, which was a key enzyme controlling fatty acid beta-oxidation and was reported to regulate muscle fuel switching between carbohydrates and fatty acids (Koves et al., 2008; Laurent et al., 2013). Moreover, knockdown of SIRT4 by adenoviral shRNA can increase the mRNA and protein content of SIRT1, thereby enhancing the expression of fatty acid oxidation genes and mitochondrial oxidation capacity in hepatocytes and myotubes (Nasrin et al., 2010) (Table 1).

## Therapeutic Targets of Hdacs and Their Potential for Metabolic Diseases

A comparative analysis used HDAC pan-inhibitor SAHA, a class I HDAC selective inhibitor (MS275) and a class II HDAC selective inhibitor (MC1568) to treat C2C12 separately. It was found that only MS275 could significantly stimulate mitochondria biogenesis and oxygen consumption (Galmozzi et al., 2013). In obese diabetic mice, it was found that specifically inhibiting Class I rather than Class II HDACs improved GTT and insulin sensitivity, increased oxidative metabolism of skeletal muscles and adipose tissue, and reduced body weight (Galmozzi et al., 2013). Similar to the effect of MS275, knockdown HDAC3 in C2C12 could also increase *Pgc-1*α, *Glut4*, *Tfam*, *Idh3*α transcription, suggesting that HDAC3 and its target genes played an important role in the above events (Galmozzi et al., 2013).

For Class II HDACs, studies have found that scriptaid, a Class IIa HDAC inhibitor, has similar effects to exercise. Six weeks of scriptaid administration significantly improved endurance performance, and significantly increased the whole-body energy expenditure and the expression of lipid oxidation related genes (*Pdk4*, *Cpt1b*, *Pgc-1*α, *Ppar*δ, etc.) of C57BL/6 mice (Gaur et al., 2016). One possible explanation of above observation is the potential target of scriptaid inhibition of titin (a structure protein of sarcomere) deacetylation and downstream genes of Class IIa HDACs (Gaur et al., 2016; Huang et al., 2019).

In mice treated with SPT1720, another activator of SIRT1, overtly enhanced endurance exercise ability was noted. Moreover, these mice were protected from HFD-induced obesity and insulin resistance, owing to an upregulation of the oxidative metabolism in skeletal muscles, liver, and BAT tissues (Pacholec et al., 2010). In order to further explore the principle of SIRT1 activation, the researchers purified SIRT1 *in vitro* and added SRT1720 and its analogs SRT2183, SRT1460, and RSV, and they found that the enzymatic activity of SIRT1 was not enhanced, suggesting that these drugs may indirectly activate SIRT1 (Pacholec et al., 2010). The enzymatic activity of SIRT1 largely depends on the content of cofactor NAD^+^. This implied that the enhancement of SIRT1 activity may be achieved through indirect upregulation of NAD^+^. Study has discovered that knocking out poly (ADP-ribose) polymerase-1 (PARP-1), which is a NAD^+^ consuming enzyme, could increase NAD^+^ content and enhance the activity of SIRT1 in BAT and skeletal muscles (Bai et al., 2011). PARP-1 inhibitors can upregulate the proteins of mitochondrial respiratory chain complexes in mice, enhance the aerobic oxidation capacity of mitochondria, and improve mitochondrial defects in the primary myotubes of obese humans (Bai et al., 2011; Pirinen et al., 2014).

Resveratrol was initially reported as an SIRT1 activator that improves mitochondrial function and exercise capacity in mice, and resists HFD-induced obesity (Lagouge et al., 2006). However, subsequent studies have discovered that administering the same dose of RSV to rats or mice does not increase mitochondrial protein content (Higashida et al., 2013). By contrast, overexpression of SIRT1 in the triceps muscle of rats decreases the mitochondrial protein content (Higashida et al., 2013). In a double-blind human trial, 11 healthy and obese men were supplied for 30-day RSV, in which the data showed that RSV can improve systolic blood pressure and homeostasis model assessment (HOMA) index-indicating glucose metabolism ability, simulating the effect of CR (Timmers et al., 2011). However, some researchers found that the overexpression of SIRT1 alone cannot mimic the CR effect in transgenic mice, and the transcriptomic changes in various tissues were quite different or even opposite (Boutant et al., 2016). Such an opposite situation may explain that the genetic model and compound stimulation are not completely consistent. RSV as a potential metabolic syndrome treatment drug still needs large-scale population sample verification.

## Conclusion

The very first mammalian histone deacetylase HDAC1 was cloned and isolated by Taunton et al. (1996), and 15,000 articles about HDACs have been published in the last 20 years. Currently, we know at least 18 HDAC proteins. They are responsible for eradicating epigenetic modifications, establishing an epigenetic off chromatin state, and regulating heritable gene expression (Yang and Seto, 2008; Haberland et al., 2009). In these processes, each HDACs may play a different role (Table 1). What kind of gene/protein is the specific downstream target of these HDACs? Is its enzyme activity related to intracellular localization and the formation of multi-protein complex? It is still one of the key and difficult issues in this field. Based on previous research experience, some techniques may be used to further experiments as follows: (1) HDACs interacting proteins/complex co-IP; (2) Protein-acetylation western; (3) Histone acetylation target gene ChIP; (4) High through put proteomics/acetylome with specific HDACs inhibitor, etc.

Histone deacetylases are potential therapeutic targets, and clinical drugs such as SAHA (Vorinostat) and FK228 (romidepsin) have been used for antitumor treatment (Bolden et al., 2006). However, they have two disadvantages: (1) great toxic and side effects and (2) difficulty in specific inhibition of HDACs activity. With the development of computer simulation technology and structural biology, it is believed that more specific HDACs inhibitors/activators can be constructed. And researches should attach importance to HDACs regulatory factors like PARP-1 when direct targeting on HDACs fails to show its effects. Further development of their inhibitors with more specificity, and trials for the treatment of metabolic diseases, may have great potential as well. Additionally, with the further development of biotechnology, some RNA therapies presented by AAV can specifically inhibit/overexpression of certain protein in skeletal muscles tissue, which may be able to target HDACs and treat related diseases (Wang et al., 2005; Long et al., 2016). However, its safety and effectiveness still need further clinical trials. Future research can discover known or unknown HDACs targeted drugs for the treatment of metabolic diseases.

## Author Contributions

HT, SL, and JR designed the literature search and wrote the review. JL, RW, and PC critically analyzed and revised the manuscript. All authors contributed to the article and approved the submitted version.

## Conflict of Interest

The authors declare that the research was conducted in the absence of any commercial or financial relationships that could be construed as a potential conflict of interest.
